# How can schistosome circulating antigen assays be best applied for diagnosing male genital schistosomiasis (MGS): an appraisal using exemplar MGS cases from a longitudinal cohort study among fishermen on the south shoreline of Lake Malawi

**DOI:** 10.1017/S0031182019000969

**Published:** 2019-09-23

**Authors:** S. A. Kayuni, P. L. A. M. Corstjens, E. J. LaCourse, K. E. Bartlett, J. Fawcett, A. Shaw, P. Makaula, F. Lampiao, L. Juziwelo, C. J. de Dood, P. T. Hoekstra, J. J. Verweij, P. D. C. Leutscher, G. J. van Dam, L. van Lieshout, J. R. Stothard

**Affiliations:** 1 Department of Tropical Disease Biology, Liverpool School of Tropical Medicine, Liverpool L3 5QA, UK; 2 MASM Medi Clinics Limited, Medical Society of Malawi (MASM), 22 Lower Scalter Road, Blantyre, Malawi; 3 Department of Cell and Chemical Biology, Leiden University Medical Center, Albinusdreef 2, 2333 ZA Leiden, The Netherlands; 4 Research for Health, Environment and Development (RHED), Mangochi, Malawi; 5 Physiology Department, College of Medicine, University of Malawi, Blantyre, Malawi; 6 National Schistosomiasis and STH Control Programme, Community Health Sciences Unit, Ministry of Health, Lilongwe, Malawi; 7 Department of Parasitology, Leiden University Medical Center, Albinusdreef 2, 2333 ZA Leiden, The Netherlands; 8 Laboratory for Medical Microbiology and Immunology, Elisabeth-Tweesteden Hospital, Tilburg, Hilvarenbeekseweg 60, Tilburg, The Netherlands; 9 Centre for Clinical Research, North Denmark Regional Hospital & Department of Clinical Medicine, Aalborg University, Region Nordjylland, Denmark

**Keywords:** CAA, CCA, diagnostics, glycans, MGS, *Schistosoma haematobium*

## Abstract

We provide an update on diagnostic methods for the detection of urogenital schistosomiasis (UGS) in men and highlight that satisfactory urine-antigen diagnostics for UGS lag much behind that for intestinal schistosomiasis, where application of a urine-based point-of-care strip assay, the circulating cathodic antigen (CCA) test, is now advocated. Making specific reference to male genital schistosomiasis (MGS), we place greater emphasis on parasitological detection methods and clinical assessment of internal genitalia with ultrasonography. Unlike the advances made in defining a clinical standard protocol for female genital schistosomiasis, MGS remains inadequately defined. Whilst urine filtration with microscopic examination for ova of *Schistosoma haematobium* is a convenient but error-prone proxy of MGS, we describe a novel low-cost sampling and direct visualization method for the enumeration of ova in semen. Using exemplar clinical cases of MGS from our longitudinal cohort study among fishermen along the shoreline of Lake Malawi, the portfolio of diagnostic needs is appraised including: the use of symptomatology questionnaires, urine analysis (egg count and CCA measurement), semen analysis (egg count, circulating anodic antigen measurement and real-time polymerase chain reaction analysis) alongside clinical assessment with portable ultrasonography.

## Introduction

Schistosomiasis remains a prevalent neglected tropical disease (NTD) in low and middle-income countries of tropical and sub-tropical regions (Colley *et al*., 2014; McManus *et al*., 2018). Each year, some 200 000 deaths occur from complications of this water-borne infection which is acquired by exposure to contaminated freshwater, often during household chores, recreational activities or income-generating activities such as fishing or agriculture (Hotez *et al*., 2008; Christinet *et al*., 2016). Following World Health Assembly resolutions to control schistosomiasis, the World Health Organization (WHO) and various stakeholders have continued urging countries in endemic areas to intensify morbidity control and strive towards interruption of schistosome transmission (WHO, 2001, 2012, 2013). A key intervention strategy is preventive chemotherapy by mass drug administration (MDA) with praziquantel (Cesol, Merck), integrated alongside complimentary measures inclusive of improved sanitation and hygiene, snail control and health education. In addition, an appropriate use of point-of-care (POC) diagnostic tests in high- and low-disease transmission settings and individual targeted treatment are vital in schistosomiasis control (Le and Hsieh, 2017).

## Diagnosis of urogenital schistosomiasis

First reported by Theodor Bilharz in 1851, infection with schistosome blood flukes gives rise to schistosomiasis, with *Schistosoma haematobium*, as recognized today, responsible for urogenital schistosomiasis (UGS); here, adult female worms, as typically found in the vesicle plexus of the bladder, produce copious amounts of eggs each day that perforate and damage various internal organs (Gryseels *et al*., 2006; Rollinson *et al*., 2013). Schistosome eggs either cross the bladder wall to be voided in the urine, or become tissue-trapped in the lower abdominal organs, inclusive of the internal and external genitalia in both genders (Ross *et al*., 2002). These incite local bleeding and induce fibrotic lesions leading to severe complications across the urogenital system. Early diagnosis in communities triggers appropriate praziquantel treatment campaigns and is paramount to maximizing the public health impact of preventive chemotherapy (Stothard *et al*., 2013).

A range of parasitological, immunological and molecular methods have been used for the detection of UGS (Stothard *et al*., 2014). The operational gold standard of diagnosing UGS is direct microscopy of filtered urine (Peters *et al*., 1976), which has been widely used in high-transmission areas to estimate the morbidity upon enumeration of eggs in 10 ml of urine (i.e. ⩾50 eggs per 10 m). However, it lacks diagnostic sensitivity in light infection when the number of eggs shed in urine is very few (counting less than one egg in 10 ml) and repeated urine samples may need to be inspected. Other less expensive methods that can complement this test include the use of questionnaires in high-risk areas for the recognition of macrohaematuria (presence of red urine), and urine reagent test strips for microhaematuria as a diagnostic indicator and marker of bladder pathology, although these suffer from poor rates of sensitivity (<75%) and cannot detect sub-clinical or acute infections (Stothard *et al*., 2014; Le and Hsieh, 2017).

Antibody-based tests such as enzyme-linked immunosorbent assay (ELISA) for IgG titres against schistosome soluble egg antigen present in human serum have been widely used for diagnosis (Le and Hsieh, 2017). Serological methods have much higher sensitivities than filtration and microscopy, especially in travellers originating from non-endemic regions, however they cannot distinguish active from past infections, nor discriminate between species of schistosome. Alternative highly sensitive approaches based on the detection of schistosome glycan antigens in blood or urine have been developed to diagnose active *Schistosoma* infections (Utzinger *et al*., 2015; Le and Hsieh, 2017), which are described below. Nucleic acid amplification tests (NAAT) have increasingly been used as highly sensitive and specific diagnostic tools, utilizing several clinical specimens (i.e. stool, urine or tissue biopsy) in diagnosing the infection (Utzinger *et al*., 2015). Currently NAAT are not widely available, owing to the need for skilled personnel, laboratory equipment and infrastructure which make roll out, especially in endemic areas, limited. Newer approaches based on loop-mediated isothermal amplification, POC magnetic bio-capture probes and microfluidic devices are being developed for resource poor settings (Minetti *et al*., 2016; Candido *et al*., 2018; Poulton and Webster, 2018).

## Glycobiology of schistosome antigens and their applications

**Fig. 1.**
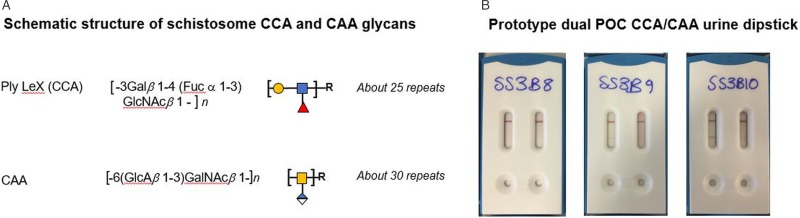
(A) Schematic outline of the chemical and polymeric glycan structures of the two most common schistosome glycoproteins (CCA and CAA) using in rapid urine-antigen detection dipsticks. (B) An illustration of future developments in POC diagnostics with a prototype dual antigen urine-dipstick detecting each antigen separately (LHS CCA, RHS CAA). Having a dual design could detect and differentiate urogenital and intestinal schistosomiasis co-infection simultaneously, however, this prototype has inadequate sensitivity for the detection of urine-CAA and needs reformulation.

A number of different schistosome antigens are excreted and secreted into the human circulation, namely cercarial antigens, gut-associated antigens from living juvenile and adult worms and antigens secreted from eggs (van Lieshout *et al*., 2000). Most of the described circulating genus specific antigens in humans are from gut-associated tissues of feeding worms, namely circulating cathodic antigen (CCA) and circulating anodic antigen (CAA). Both CCA and CAA, see Fig. 1A, are detectable in the host's serum as well as in urine although relative concentrations can differ and these are thought to have immunomodulating effects within the parasitized host (Dam and Deelder, 1996; van Dam *et al*., 1996; van Diepen *et al*., 2012; Hokke and van Diepen, 2017).

Glycoproteins containing CCA are produced by the gut epithelium of schistosomes presumably for its protection and are regurgitated into the human bloodstream upon digestion of the blood meal as worms have a blind-end gut. The structure of these positively-charged antigens consists of multiple trisaccharide units (Lewis-X) containing fructose, galactose and N-acetyl-galactosamine, Fig. 1. In addition, CCA epitopes are also present on *Schistosoma* egg secretions. They also evoke high titres of specific IgM/IgG antibodies, which may be responsible for the mild-moderate neutropenia during schistosome infection (van Dam *et al*., 1996).

Making use of CCA, POC urine-based lateral-flow assays have been developed since the late 90s and have been commercially available since 2002 in the form of reagent dipsticks or cassettes, with carbon- or gold-labelled monoclonal antibodies and interpreted visually (van Dam *et al*., 2004; Le and Hsieh, 2017). Detectable CCA-levels typically correlate with active schistosome infection, which become undetectable after successful praziquantel treatment. However, upon comparison with intestinal schistosomiasis (caused by *Schistosoma mansoni*) these tests perform poorly for UGS see Table 1, hence combining it with urine filtration is needed, and can help with simultaneous detection of co-infected cases (i.e. *S. haematobium* and *S. mansoni*). Since the first use of point-of-care circulating cathodic antigen (POC-CCA) tests, they have been subject to many evaluations of their performance, with the WHO now endorsing these tests as appropriate for estimating prevalence thresholds for intestinal schistosomiasis to guide preventive chemotherapy (Colley *et al*., 2017; Bärenbold *et al*., 2018). Current developments in POC testing include a prototype dual antigen cassette with both CCA and CAA strips included, enabling the detection and discrimination of intestinal and UGS simultaneously see Fig. 1B (see https://freebily.eu/about/).

**Table 1.** Sensitivity and specificity of urine POC-CCA tests to diagnose *S. haematobium* infection, in comparison to urine filtration and microscopy as a routine standard test

Source	Prevalence (%)	Sensitivity (%) (95% CI)	Specificity (%) (95% CI)
Stothard *et al*. (2009)	31	9 (2–21)	98 (93–100)
Ayele *et al*. (2008)	Moderate	52 (42–62)	64 (54–73)
Midzi *et al*. (2009)	Moderate	79 (70–86)	44 (36–52)
Ashton *et al*. (2011)	Moderate	37 (26–49)	79 (72–84)
Obeng *et al*. (2008)*	Not stated	41 (not stated)	91 (not stated)
El-Ghareeb *et al*. (2016)*	5	88 (not stated)	96 (not stated)
Sanneh *et al*. (2017)*	23	48 (not stated)	76 (not stated)
Rubaba *et al*. (2018)*	40	68 (not stated)	46 (not stated)
Range	–	9–88	44–98

Data adapted from Ochodo *et al*. (2015), where intensities of infection are classed as ‘moderate’. Additional sources marked by ‘*’. Where data is missing this is marked as ‘not stated’.

The alternative CAA antigens are also gut-associated glycoproteins but are negatively charged. The structure of CAA is made up of carbohydrate chains which consist of multiple disaccharide units containing N-acetyl-galactosamine and glucuronic acid (Fig. 1). It binds to the collagen-like stalk of first complement component C1q, probably preventing host complement from attacking the schistosome gut (van Dam *et al*., 1993). CAA is also present in urine or serum of actively infected people as shown by monoclonal antibody-based antigen detection ELISA's and more recently up-converting phosphor-lateral flow assay (UCP-LF CAA) (Corstjens *et al*., 2008). This assay has shown to be more sensitive and specific especially in low-transmission areas but is unable to differentiate between urogenital and intestinal schistosomiasis (Corstjens *et al*., 2015; Knopp *et al*., 2015). The UCP-LF-CAA test therefore has future application in the general monitoring of schistosomiasis as disease control programmes move towards interruption of transmission or endgame scenarios (Corstjens *et al*., 2017; Stothard *et al*., 2017).

## Focus on male genital schistosomiasis

Despite being known as urogenital disease, genital manifestations of *S. haematobium* infections in both genders have been underreported, ignored and less frequently diagnosed. Unlike the increasing awareness of female genital schistosomiasis (FGS) within endemic populations (Christinet *et al*., 2016), in part due to the wider health-seeking behaviour of women, an appreciation of male genital schistosomiasis (MGS) remains limited. MGS describes a specific manifestation associated with the presence of schistosome eggs in seminal fluids and genital tissues in addition to various pathologies in the male genital system (WHO, 2018). Following its first description by Madden, (1911), several research studies and case reports have described the condition, unknowingly present in endemic areas, causing genital and pelvic pain, haemospermia, abnormal ejaculates, infertility among other abnormalities, also detectable by radiological methods (Vilana *et al*., 1997; Squire and Stothard, 2014; Kayuni *et al*., 2019). Furthermore, studies have shown increased levels of inflammatory cells and immunological mediators in semen harbouring *Schistosoma* eggs which necessitate HIV attachment and replication, and changes in seminal viral loads among co-infected males, highlighting the plausible link of increased risk of HIV transmission from co-infected males to their sexual partners (Leutscher *et al*., 2000, 2005, 2008*b*; Stecher *et al*., 2015; Midzi *et al*., 2017).

Substantial progress has been made in developing a gold standard technique for a definite FGS diagnosis, namely colposcopy in gynaecological clinics (WHO, 2015) often with genital tissue biopsy for histopathology in the hospital laboratory. Conversely, MGS remains largely undefined, an orphan within disease syndromic triage (Kayuni *et al*., 2019). At present, semen microscopy is considered as a standard technique for diagnosing active MGS infection and assessing its severity, since the schistosome eggs are directly visualized. Urine filtration has been used as diagnostic proxy markers in the presence of MGS symptoms; however, there have been reports of seminal schistosome eggs in urine negative patients (Schwartz *et al*., 2002; van Delft *et al*., 2007). In addition, genital tissue biopsy and ultrasonography can be applied as diagnostic tools relevant in diagnosing MGS through observation of pathologies associated with the disease in the absence of other genital diseases, which have successfully been studied and reported (Leutscher *et al*., 2008*b*).

In light of the above, we describe the research study protocol of our longitudinal cohort MGS study among fishermen (with and without HIV infection) along the south shoreline of Lake Malawi in the Mangochi District. Preliminary results of the study at baseline are presented together with two exemplar clinical case reports, illustrating the diagnostic challenges for MGS.

## Longitudinal cohort study of MGS along southern Lake Malawi shoreline

Malawi is one of the South Eastern African countries where both *S. haematobium* and *S. mansoni* are prevalent and highly focal around most water bodies (Teesdale and Chitsulo, 1985; Makaula *et al*., 2014). The shoreline of Lake Malawi, the third largest lake in Africa, is endemic for urogenital schistosomiasis, with a high prevalence of urine-ova patent *S. haematobium* infections (Madsen *et al*., 2011; Stauffer *et al*., 2014). More recently, with the discovery of *Biomphalaria pfeifferi* there is also emergence of autochthonous transmission of intestinal schistosomiasis (Alharbi *et al*., 2019). More broadly, children, women, farmers and fishermen are at greater risk of the disease due to more frequent water contact.

The prevalence of HIV in Malawi is considered high (10.6%), especially in this lakeshore region (11.8%), despite the control efforts contributing to reducing the incidence and mortality (NAC, 2015; UNAIDS, 2016). Despite the wide awareness for the significant burden of UGS in the area, MGS typically remains undiagnosed and underreported among men. With no information about the burden of MGS on the south shoreline of Lake Malawi in Mangochi District, our research study set out to determine the current prevalence and morbidity of MGS among local fishermen on the shoreline and the potential risk of raised HIV transmission through viral load shedding in semen.

## Study methodology

### Study area, population and sampling

The research study was conducted among fishermen living in fishing communities (villages) identified and selected along the south shoreline of Lake Malawi in Mangochi District from October 2017 to December 2018. Mangochi is the largest district in the southern region of Malawi, covering 6729 km^2^ of land with at least 1.1 million people (NSO, 2018). The district has a tropical continental climate with a longer dry season of cold weather from May to August and hot weather from September to November, and a relatively shorter wet season from December to April (NSO, 2011). Most fishermen in the area live in specific fishing villages, closer to the lake to carry out their routine fishing related activities.

This was a longitudinal cohort study, comprising baseline surveys of MGS among fishermen and follow-up studies after praziquantel treatment, conducted in villages and nearby health centres. Fishermen aged ⩾18 years willing to provide written informed consent were eligible to participate in the study. Using the estimated 20% prevalence of *S. haematobium* in adults from previous studies and assuming 10% having MGS, a minimum sample size of 275 fishermen (adjusted for assumed 10% loss to follow-up), was planned to be randomly selected for the study to measure the current prevalence of MGS and subsequent follow-up studies (Kirkwood and Sterne, 2006; CDC, 2014).

### Data collection and analysis

The following are the data collection methods and analyses that were used in the study:

#### Individual questionnaires

After briefing about the study and obtaining written informed consent, fishermen were recruited in their communities and interviewed with individual questionnaires, collecting information on demographic, health, hygiene, sanitation and socio-economic characteristics. This information assessed their knowledge, perceptions, attitudes and practices on MGS and HIV. The questionnaires were developed from standardized questions administered elsewhere in a similar study (Ukwandu and Nmorsi, 2004). The questionnaires were piloted on the first 10 participants to assess the reliability of the questions. After the questionnaire interviews, the participants were invited to the nearby health facility to submit urine, semen and for ultrasonography examination.

#### Parasitological analyses

**Fig. 2.**
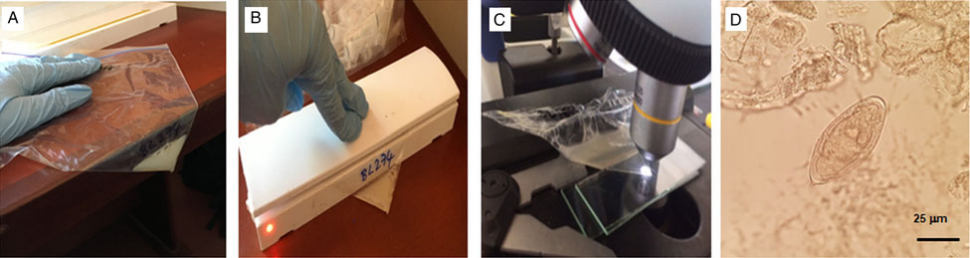
(A) pictorial methodology of visualization of schistosome ova in semen with a clean, non-sterile transparent plastic bag. (A) Semen is concentrated to one corner; (B) the bag is heat sealed to trap the liquid; (C) the bag is placed on microscope stage and inspected at ×40 or ×100 magnification; (D) an egg of *S. haematobium* with miracidium inside.

At the health facility, they were provided with a clean sample container to submit urine, between 10 am and 2 pm for filtration to examine for schistosome eggs (confirming UGS). Semen was submitted in a clear, transparent, self-sealing plastic bag, see Fig. 2, after abstaining from coitus for two days to examine for MGS, defined in the study as the presence of schistosome eggs in semen.

*Urine analysis – filtration:* Urine was analysed immediately for macrohaematuria by visual inspection using a urine colour card, and then for microhaematuria, leukocytes and proteinuria using reagent strips (Siemens multistix 10G) and scores were recorded in the following categories: negative, trace, +, ++ and +++. The POC-CCA test was conducted on the urine to assess for possible intestinal infection by *S. mansoni*, following manufacturer's instructions (Rapid Medical Diagnostics, South Africa; batch no. 171103130) and as described previously (van Dam *et al*., 2004). Urine was measured and recorded accordingly, before conducting filtration following approved standard guidelines (WHO, 1991; Cheesbrough, 2009).

The entire volume of urine was filtered through a disinfected filter containing a clean polycarbonate membrane with 20 *µ*m pores to trap all *S. haematobium* eggs in the sample. The membrane was removed, placed on a standard glass slide and examined under a microscope. Iodine was added to visualize the eggs distinctly. The number of eggs was calculated by first, dividing the total eggs observed by the total volume filtered and then multiplying by 10. The resultant egg count was recorded per 10 ml of urine. Highest infection intensity for UGS was defined as egg count of ⩾50 eggs per 10 ml urine as widely described (Cheesbrough, 2009).

*Seminal microscopic analysis:* After submission, the bag with semen was placed at room temperature on a clean bench surface to allow the semen to liquefy. Thereafter, the semen was pushed gently to one corner of the clear plastic bag. Then the bag was heat-sealed to evenly concentrate the semen for easy visualization during microscopy. The direct examination of the semen bag was conducted under a microscope to check for schistosome eggs and the presence of leukocytes (WHO, 2010), thereafter the results were recorded as per ml of ejaculate.

Afterwards, the semen was measured and centrifuged at 3000 rpm for 5 min to collect the seminal plasma. The sediment was re-dissolved in 0.5 ml normal saline for wet mount inspection using 2–3 drops and placed on a slide with a coverslip for microscopy, followed by recording of the results. Thereafter, 0.5 ml of ethanol was added to the remaining sediment for preservation and stored together with the seminal plasma at −80 °C in preparation for shipment to the United Kingdom for the real-time polymerase chain reaction (PCR) analysis of *Schistosoma* genus DNA and HIV viral load for those participants on antiretroviral therapy (ART).

*UCP-LF CAA seminal analysis:* A trichloroacetic acid (TCA) extraction was performed on the seminal plasma following standard methods used for serum with an equal volume of 4% w/v TCA (Corstjens *et al*., 2008). Small volume extraction (50 *µ*l seminal plasma with 50 *µ*L TCA) in microfuge tubes resulted in a clear supernatant after centrifugation (5 min, 13 000 rpm). UCP-LF CAA analysis was performed according to standard methods with 20 *µ*l of the clear supernatant, with a cut-off threshold of 10 pg ml^−1^. High-volume extraction (0.5 ml seminal plasma with 0.5 ml TCA and a cut-off threshold of 1 pg ml^−1^) required extended centrifugation time (30 min) before a clear supernatant was obtained; the resulting pellet was not rigid. Amicon 10 kDa centrifugal filtration devices (Merck Millipore) were used to concentrate 0.5 ml of the clear supernatant targeting concentration to 20 *µ*l following standard methods used for serum undergoing centrifugation for 30 min at 13 000 rpm (Corstjens *et al*., 2014).

Schistosoma *DNA real-time PCR analysis:* The ethanol preserved semen sediment was defrosted and centrifuged for 1 minute at 10 000 rpm. The ethanol layer was removed, and the pellet washed twice with 1 ml of phosphate buffered saline (PBS). The pellet was suspended in 0.4 ml of PBS containing 2% polyvinylpolypyrrolidone (Sigma, Steinheim, Germany). The suspension was heated for 10 min at 95 °c and stored frozen overnight at −20 °C. DNA was extracted using the QIA symphony DSP virus/pathogen midi kit and pathogen complex 400 protocol of the QIA symphony Sample Processing (SP) system (Qiagen, Hilden, Germany). In each sample, a fixed amount of Phocine Herpes Virus 1 (PhHV-1) was added within the isolation lysis buffer, to serve as an internal control for the isolation procedure and to monitor the inhibition of the real-time PCR. The *Schistosoma* genus-specific real-time PCR was performed using primers and probes as described previously (Obeng *et al*., 2008; Kenguele *et al*., 2014).

#### Ultrasonography examination

Participants were briefed on the transabdominal and scrotal ultrasonography procedures to be conducted on them using a portable Chison Q5 ultrasound scanner with 3.5 MHz probe supplied by Mount International United Services Ltd, Gloucester, United Kingdom. Participants were asked to present with a full bladder, before the procedure to increase the visualization and validity of the images. The participant was positioned supine on the examination couch with the scanner set up on their right side. Whenever possible, room lightning was turned off to maximize screen visibility.

The scanning procedure investigated the appearance, size and abnormalities of the following key pelvic and genital organs: urinary bladder (shape, thickness, calcifications, masses, polyps), seminal vesicles (symmetry, thickness, nodules, echogenicity) and scrotum (tests, epididymis: nodules, masses, calcifications, hydroceles), according to evidence-based recommendations (Vilana *et al*., 1997; WHO, 2000; Martino *et al*., 2014). The observations made during the procedure and degree of visualization were recorded accordingly.

All clips and images were stored on the device before transferring to the external hard drive for further analyses. A sample of 15% of the scan images were randomly selected and re-read by specialist radiologist for quality control, who conducted training of the study scanning personnel. All participants were notified of pathological findings that day, and further appropriate investigations and management were organized in accordance with standard clinical practice. Thereafter, praziquantel treatment at 40 mg kg^−1^ as a single dose was offered along with an invitation to follow-up studies after 1-, 3-, 6- and 12-months.

#### Data analyses

All the information collected during the study was screened and quality-controlled before entry into Microsoft Excel and SSPS computer packages. Screening for errors and cleaning were conducted, before commencing statistical analyses to present the results of the study.

*Ethical considerations:* Ethical clearance for the study was granted by both the Liverpool School of Tropical Medicine Research Ethics Committee (LSTM REC Approval number: 17-018) and the National Health Sciences Research Committee of Malawi (NHSRC Approval number: 1805). Utmost privacy and confidentiality were maintained in the study and where necessary, the information was anonymized to protect the identity of the participant. Participants were informed of their right to opt-out at any stage of the study if they wish to do so. No disruption was caused to their normal daily activities or seeking other services at any health facility. Since this was a test-to-treat study, participants were offered praziquantel treatment at the end of the visit before inviting them to the next follow-up study.

## Preliminary baseline results of the study

A total of 376 fishermen were recruited at baseline into the study who were interviewed with questionnaires, 56 were HIV infected and receiving ART. The participants came from 39 villages located in two Traditional Authorities (T/A) of Mponda and Nankumba, along the shoreline within the study area. The median age of the participants was 30.0 years with a range of 18.0 to 70.0 years (interquartile range [IQR]: 13.0) and their duration of stay in the fishing village ranged from 1 month to 70 years (median: 20.0 years; IQR: 24.3). The mean weight of the participants was 59.1 kg (range: 43.0–85.0 kg; 95% C.I.: 58.1–59.9).

Out of the total recruited participants, only 210 submitted urine after questionnaires (55.9%) and 114 submitted semen (30.3%). Urine reagent dipstick showed that most of the urine was observed to be negative for leukocytes (82.4%), blood (72.9%), protein (63.8%) and glucose (100%). After urine filtration, 36 participants (17.1%) had *S. haematobium* eggs in urine (UGS), their mean egg count was 14.8 eggs per 10 ml and ranged from 0.1 to 186.0 eggs (median: 0.9, IQR: 5.4). The total urine volumes ranged from 10 ml to 240 ml and only three participants had the highest infection intensity (92, 137.8 and 186 eggs). Eight (3.8%) had a positive POC-CCA test, suggestive of possible *S. mansoni* intestinal schistosomiasis infection.

For those who submitted semen, 12 (10.4%) had *S. haematobium* eggs in semen (MGS). The median egg count was 2.9 per ml of ejaculate, ranging from 0.4 to 30.0 eggs and the volume of semen ranged from 0.1 to 4.5 mL (median: 1.4 ml). The semen bag method identified eight participants (66.7%) whose median egg count was 0.8, while the centrifuge method identified nine participants (75.0%) with a median of 2.9 eggs, and only five participants (41.7%) were observed to have MGS by both methods simultaneously. Eight participants (66.7%) with MGS had no eggs in urine, the median volume of which was 60.0 ml (range: 10–90 ml). Upon interview on the use of the collection bag, 71.0% (*n* = 51) men preferred the use of a bag method *vs* the use of the screw top sample container.

Transabdominal and scrotal ultrasonography was conducted on 125 participants at baseline and 25 abnormalities were noted in the gential organs. For UCP-LF CAA analyses of the first 14 semen samples, only five samples were noted to generate a supernatant that could be concentrated using the Amicon concentration devices. Further analyses are underway for the remaining samples collected in the study.

The real-time PCR conducted on 65 semen samples revealed that 18 (26.5%) were positive (Ct-value range: 18.6–36.6) of which seven had no eggs in semen or urine while only six participants had eggs in urine only. For those participants with eggs in semen but negative on the PCR could explain an old infection with dead eggs which were migrating in the genital organs and then released into ejaculatory ducts and seminal fluid. Using the seminal schistosome PCR as a reference test for MGS diagnosis in comparison with semen microscopy, the latter had a sensitivity of 25.3% and a specificity of 70.6%, which is substantially lower than using urine filtration as a proxy for MGS diagnosis. Interestingly, the positive predictive value of semen microscopy was 40.6% while the negative predictive value was 77.3%, which highlights the further need to develop more sensitive and specific diagnostic tests to diagnose MGS.

Here we present two exemplar clinical case reports, summarized in Table 2, demonstrating the outcomes and potential challenges of different diagnostic tests for MGS used in the longitudinal study.

**Table 2.** Summary of the clinical cases from the longitudinal cohort research study on MGS among local fishermen along south shoreline of Lake Malawi

Study time-point	Parameter	Case 1	Case 2
Baseline	Symptoms/diseases experienced	Scrotal pain	No
	Previous MDA	No	No
	Urine		
	Volume (ml)	70	35
	Appearance	Clear	Clear
	Reagent dipstick	Trace (L, P) Positive + + + (B)	Trace (P) Negative (L, B)
	POC-CCA	Negative	Positive
	Filtration (eggs per 10 ml)	21.4	0
	Semen		
	Volume (ml)	2.0	2.5
	Semen bag (eggs per ml)	2.5	0
	Centrifuge (eggs per ml)	6.5	0
	UCP-LF CAA (pg ml^−1^)	5	0
	Real-time PCR (Ct value)	26.6	Negative
1-month Follow up	Urine		
	Volume (ml)	45	100
	Appearance	Clear	Clear
	Reagent dipstick	Positive + + + (L, P) Negative (P)	Negative (L, B, P)
	POC-CCA	Negative	Negative
	Filtration (eggs per 10 ml)	2.4	0
	Semen		
	Volume (ml)	2.5	0.5
	Semen bag (eggs ml^−1^)	0	0
	Centrifuge (eggs ml^−1^)	0	0
	UCP-LF CAA (pg ml^−1^)	N/A	0
	Real-time PCR (Ct value)	Negative	Negative
3-months Follow up	Urine		
	Volume (ml)	95	100
	Appearance	Clear	Clear
	Reagent dipstick	Positive + + (B), + (P), trace (L)	Negative (L, B, P)
	POC-CCA	Negative	Negative
	Filtration (eggs per 10 ml)	19.7	0
	Semen		
	Volume (ml)	2.6	2.0
	Semen bag (eggs per ml)	0	0
	Centrifuge (eggs per ml)	0	0
	UCP-LF CAA (pg ml^−1^)	N/A	0
	Real-time PCR (Ct value)	28.8	Negative
6-months Follow up	Urine		
	Volume (ml)	N/D	100
	Appearance	N/D	Clear
	Reagent dipstick	N/D	Negative (L, B, P)
	POC-CCA	N/D	Negative
	Filtration (eggs per 10 ml)	N/D	0
	Semen		
	Volume (ml)	N/D	0.8
	Semen bag (eggsper ml)	N/D	0
	Centrifuge (eggsper ml)	N/D	0
	UCP-LF CAA (pg ml^−1^)	N/D	1
	Real-time PCR (Ct value)	N/D	Negative
12-months Follow up	Urine		
	Volume (ml)	N/D	100
	Appearance	N/D	Clear
	Reagent dipstick	N/D	Trace (L), negative (B, P)
	POC-CCA	N/D	Trace
	Filtration (eggs per 10 ml)	N/D	0
	Semen		
	Volume (ml)	N/D	1.1
	Semen bag (eggs per ml)	N/D	0
	Centrifuge (eggs per ml)	N/D	0
	UCP-LF CAA (pg ml^−1^)	N/D	N/A
	Real-time PCR (Ct value)	N/D	31.2

Urine reagent dipstick test result: L, leukocytes; B, blood; P, protein; N/A, result not available, test currently underway; N/D, participant not available, test not done.

## Case 1

This concerns a 24-year-old HIV-negative fisherman recruited into the study, 48.0 kg body weight, had been fishing in the lake for 14 years. He was experiencing occasional spontaneous pain in the scrotal region for over a month. He had no previous history of receiving praziquantel during annual MDA campaigns. After describing his symptoms during questionnaire interview, he was requested to submit urine and semen samples for parasitological diagnosis of egg-patent *S. haematobium* infections upon microscopy.

His 70 ml mid-morning urine was normal in colour, and the urine reagent strip showed a trace of leukocytes and protein, the presence of microhaematuria (+++ blood score), while the POC-CCA test was negative. After filtration, 150 *S. haematobium* eggs were detected on microscopy (21.4 eggs per 10 ml of urine). He submitted 2 ml of semen in which 5 eggs were observed by the bag method and 13 eggs after centrifugation (6.5 eggs per ml of ejaculate), however, no leukocytes were observed.

**Fig. 3.**
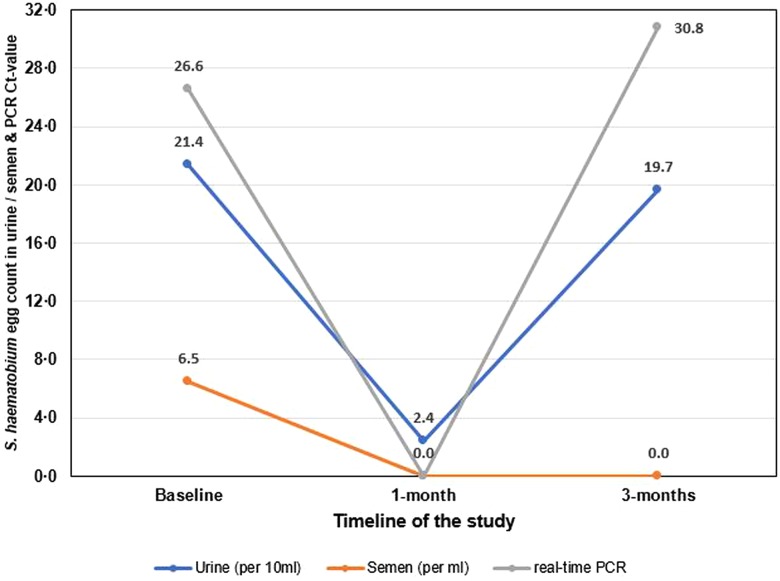
A line graph of the clinical Case 1 in the longitudinal cohort study showing results of *S. haematobium* egg counts in urine (per 10 ml) and semen (per ml); and Ct-values for real-time PCR analysis of semen at baseline, 1- and 3-month follow-up studies.

A CAA concentration of 5 pg ml^−1^ was found in the seminal plasma on UCP-LF CAA analysis, using the high-volume extraction procedure. Analysis of DNA extracted from the harvested semen sediment registered a strong positive output upon real-time PCR (Ct-value: 26.6). The results of the egg count and real-time PCR at baseline and follow-ups are shown in Fig. 3.

Transabdominal and scrotal ultrasonography showed a thickened bladder wall (⩾11 mm) and asymmetrically enlarged seminal vesicles (⩾15 mm). Case 1 was treated with praziquantel tablets and examined after 1 month and 3 months, where no schistosome eggs were detected in semen on both occasions. The real-time PCR on the semen sediment was negative at 1 month but became positive again at 3 months, the Ct-value was 30.8. A total of 11 and 187 schistosome eggs were observed in his urine at 1- and 3-months respectively (2.4 and 19.7 eggs per 10 ml of urine), with normal findings on ultrasonography.

## Case 2

A 26-year-old HIV-negative fisherman recruited into the study, 59.7 kg body weight, had been fishing in the lake one day in a week for 11 years. He didn't report any symptoms or illness in the preceding months or receiving praziquantel during annual MDA campaigns.

He submitted 35 ml mid-morning urine, which was normal in colour, and the urine reagent strip showed a trace of protein, no leukocytes or blood, and the POC-CCA test was positive. No schistosome eggs were detected on urine filtration nor in his 2.5 ml ejaculate, which had no leukocytes. The real-time PCR analysis of his semen was negative, transabdominal and scrotal ultrasonography examination were normal and he was then given praziquantel treatment. Follow-up at 1-, 3-, 6- and 12-months showed negative POC-CCA results, no schistosome eggs in urine or semen, negative real-time PCR and normal ultrasonography examinations, except a seminal plasma CAA concentration of 1 pg ml^−1^ on UCP-LF CAA analysis at 6-months, Trace on POC-CCA and positive real-time PCR of 31.2 on his semen at 12-months.

## On the detection of MGS

To our knowledge, this is a first longitudinal cohort research study and case description of MGS among local fishermen living along south shoreline of Lake Malawi, a schistosomiasis-endemic region in the South Eastern part of Africa. Previous studies have focussed on travellers and expatriates visiting the lake for recreation and sports. In this endemic setting of Malawi, it is clear that MGS remains an unrecognized, undiagnosed and underreported illness among fishermen, despite the more obvious burden of urogenital schistosomiasis, and we intend for our longitudinal cohort study to stimulate growing research interest into this condition. Our Case 1 presented symptoms of MGS previously described in the literature, resulting from granulomata and associated pathologies caused by schistosome eggs during their migration and entrapment through the walls of internal genital organs before being released in semen, which is pathognomonic of MGS. However, these symptoms are commonly mistaken for sexually transmitted infections, which result in poor diagnosis and management of this treatable and preventable condition. Praziquantel was generously donated and distributed to over 89 million people in 2016 through MDA campaigns although the focus is upon treatment of school-aged children rather than fishermen (Leutscher *et al*., 2008*a*; Yirenya-Tawiah, Ackumey and Bosompem, 2016; WHO, 2018).

Our cases showed positive results on the UCP-LF CAA analysis on semen which was performed for the first time in its development, hence highlighting the need to optimize the semen sample concentration technique to allow better detection of CAA concentrations below 10 pg ml^−1^. Our case illustrated a downward trend in egg count after praziquantel treatment at 1-month as well as a negative real-time PCR result, showing clearance of eggs in semen and a putative cure of *S. haematobium* infection. This demonstrates that praziquantel appears effective at the standard dose for MGS treatment, though repeated or increased doses could be beneficial in cases of heavy infestation and to counter reinfection (Schwartz *et al*., 2002), as was seen in this case. It is worthy to note that at 3 months his urine egg-count increased together registering a positive real-time PCR result indicative of a newly acquired infection. To mitigate re-infection, other strategies such as public health education on avoidance of high-risk bathing areas, earlier diagnosis and stepped-up treatment of regularly patently infected people are needed, especially to regress any progressive morbidity.

Of particular interest is the only positive POC-CCA test in case 2 while all the other diagnostic tests were negative for schistosomiasis. This points towards the strong possibility of intestinal schistosomiasis caused by *S. mansoni*, as was recently discovered during the course of our longitudinal study (Alharbi *et al*., 2019), which redefines the epidemiology of the disease along the shoreline. The negative POC-CCA result on follow up studies after praziquantel may further allude to the fact that this infection was cleared, owing to satisfactory cure rates, previously reported (Knopp *et al*., 2013). Definitive results of this case could arise from further additional diagnostic tests for intestinal schistosomiasis and possible schistosome hybrids recently reported in the area (Webster *et al*., 2019).

Chronic pathologies in seminal vesicles, vas deferens, testes and prostrate alongside the urinary bladder such as calcifications, hyper-echogenicity, organ enlargements and among others can be observed on ultrasonography (Vilana *et al*., 1997; Ramarakoto *et al*., 2008). This provided key morbidity features aiding the MGS diagnosis and management, although this procedure is seldom available in most rural health facilities in Malawi. Our case 1 displayed similar MGS abnormalities in such organs described in previous publications (Corachan *et al*., 1994; Vilana *et al*., 1997), and showed some resolution after praziquantel therapy. Where such services are available, appropriate usage of such services could further aid in MGS management and avoid unnecessary invasive procedures which continue to be implored and reported.

Apart from ultrasonography, diagnosis of MGS using a gold standard technique of semen examination remains a substantive challenge among medical professionals as it requires the cumbersome task of semen collection using sterile specimen containers or non-spermicidal condoms which are costly and unavailable in endemic areas including Malawi (WHO, 2010; Kipandula and Lampiao, 2015). In addition, most facilities have poorly equipped laboratories, with limited-trained personnel to prepare and examine semen, hence the design of our research study to use a low-cost, clear self-sealing plastic bag to quickly and easily examine the semen by direct microscopy can improve opportunities for the diagnosis of MGS in resource poor settings.

The plastic bag is a readily available commodity used for various activities in households, workplaces and health facilities and is more affordable (costs 5 cents) than the sterile container or non-spermicidal condom (75 and 30 cents, respectively). Our Case 1 showed similar results between the bag method and the standard method of centrifugation. In addition, the preference for using the bag in comparison with the screw top sample container by participants, suggest the need for further validation of this method to determine its applicability in clinical practice, especially in limited-resource settings.

The real-time PCR on the harvested semen showed an increase in the prevalence of MGS (data not shown) which highlights the need to develop more sensitive and specific diagnostic tests to diagnose MGS. This is especially important since the current low-cost standard technique misses a substantial number of individuals at high-risk of the infection. Although urine filtration is used as a proxy for MGS diagnosis, its lower sensitivity in individuals with light infections and also in low-transmission areas, emphasizes the need to improve the diagnostic platform for MGS.

## What outlook for MGS diagnostics?

Urogenital schistosomiasis remains a prevalent NTD in low- and middle-income tropical countries, particularly in sub-Saharan Africa. As a consequence of MDA, the sensitivities of current urine-diagnostic tools will further reduce and pose a future challenge in the detection of lighter infections linked with clinical disease. MGS is an important but ignored complication of UGS, and there is as of yet no single diagnostic criteria entirely satisfactory. Indeed, greater effort should be made to improve specific POC diagnostics, to complement and monitor the progress of MDA programmes and integrated control strategies. We now therefore call for an all-inclusive diagnostic algorithm for MGS to be developed that accurately identifies infected men with delivery of best treatment.
